# Tracking Interpersonal Violence: A 13-Year Review of Cases in a Referral Hospital (2009–2022)

**DOI:** 10.3390/ijerph22040607

**Published:** 2025-04-11

**Authors:** Andrés Santiago-Sáez, Montserrat Lázaro del Nogal, Patricia Villavicencio Carrillo, María Teresa Martín Acero, Cesáreo Fernández Alonso, Raquel Lana Soto

**Affiliations:** 1 Faculty of Medicine, Universidad Complutense de Madrid, Member of Instituto de Investigación Sanitaria Hospital Clínico San Carlos (IdISSC), Calle del Prof Martín Lagos, s/n, Moncloa–Aravaca, 28040 Madrid, Spain; 2 Geriatric Department, Hospital Clínico Universitario San Carlos, Commission Against Violence at Hospital Clínico San Carlos, 28040 Madrid, Spain; montserrat.lazaro@salud.madrid.org; 3 Psychic Trauma Unit of Hospital Clínico San Carlos, Faculty of Psychology, Complutense University of Madrid, 28040 Madrid, Spain; patriciaesther.villavicencio@salud.madrid.org; 4 Health Department, Hospital Clinico San Carlos, 28040 Madrid, Spain; mmacero@salud.madrid.org; 5 Emergency Service, Hospital Clinico San Carlos, 28040 Madrid, Spain; cesareo.fernandez@salud.madrid.org; 6 Internal Medicine Department, Hospital Clínico San Carlos, Commission Against Violence, Hospital Clínico San Carlos, 28040 Madrid, Spain; raquel.lana@salud.madrid.org

**Keywords:** interpersonal violence, risk factors, prevention strategies

## Abstract

Interpersonal violence involves intentional physical harm or force with psychological effects, influenced by interpersonal and societal factors. Health systems play a vital role in detecting and addressing such violence, requiring improved training and surveillance. Our hospital established a registry of suspected violence cases reported by healthcare professionals to enhance understanding, prevention strategies, and recognition of violence types and risk factors. Since 2009, all admitted patients suspected of experiencing violence were included, regardless of age or gender. Data from 2009 to 2022 covered demographics, violence details, medical interventions, and legal actions. Among 1284 patients, 83.4% were seen in the emergency department, with women comprising 80.8%, and with a mean age of 33.19 years. Reports of violence rose from 1.9% in 2009 to 16.9% in 2022. Risk factors included pregnancy [5.6%], age below 18 or over 80 [18.9%], disability [10.2%], and psychiatric conditions [11.3%]. Perpetrators were known in 56.8% of cases, mainly intimate partners [25.2%], with 29.4% of victims living with the aggressor. Doctors were primary reporters, and injury reports were issued in 65.5% of cases. Violence types included physical [44.5%], sexual [22.4%], psychological [13.3%], and economic [12.5%], with 36.3% of cases involving multiple types. Routine hospital screening and trained staff can improve victim support and enable injury prevention programs.

## 1. Introduction

Violence, as defined by the World Health Organization in the World Report on Violence and Health [WRVH], encompasses “the intentional use of physical force or power, threatened or actual, against oneself, another person, or against a group or community, that either result in or has a high likelihood of resulting in injury, death, psychological harm, maldevelopment, or deprivation” [1,2]. In this context, violence extends beyond physical injury to encompass instances where psychological harm, maldevelopment, or deprivation occurs; thus, acts of omission or neglect, not solely commission, can be categorized as violent [1].

While the terms “violence” and “abuse” are frequently used interchangeably, a distinction exists between the two. A key feature of abuse is coercive control, which involves tactics such as emotional manipulation, isolation, and financial exploitation. This form of abuse can be particularly damaging, even in the absence of physical violence [3].

The WRVH classifies violence into three categories based on the perpetrator: self-directed, interpersonal, or collective. This initial classification distinguishes between violence that an individual commits against themselves, violence perpetrated by another person or a small group, and violence carried out by larger entities such as states, organized political factions, militia groups, and terrorist organizations. Self-directed violence encompasses suicidal behavior (suicidal thoughts, attempted suicides, and completed suicides) and self-abuse including self-mutilation. Interpersonal violence is divided into (1) family and intimate partner violence, occurring usually, but not exclusively, in domestic environments, and includes child abuse, intimate partner violence, and abuse of the elderly, and (2) community violence, reflecting aggressions between unrelated persons, including youth violence, random acts of violence, rape or sexual assault by strangers, and violence in institutional settings such as schools, workplaces, prisons, and nursing homes. Collective violence is divided into social, political, and economic types, each suggesting different motivations for actions by large groups or states. Social violence includes hate crimes, terrorism, and mob actions aimed at advancing specific agendas. Political violence includes warfare and state aggression. Economic violence involves attacks motivated by financial gain, such as disrupting economic activities or creating divisions. Clearly, larger groups can be driven by multiple motives in their violent actions. Furthermore, it delineates four additional categories based on the nature of violence: physical, sexual, psychological, or involving deprivation or neglect [1].

**Figure 1:**
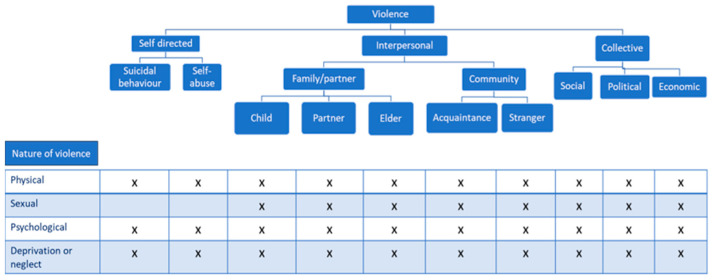
Typology of interpersonal violence [1].

Given the multifaceted nature of violence, various forms may co-occur, rendering them non-mutually exclusive [1], as shown in Figure 1.

Though not perfect and lacking universal consensus, this classification offers a valuable framework for comprehending the intricate patterns of violence occurring globally.

The International Classification of Diseases [ICD] codes, used around the world to code mortality and morbidity data, include mechanism of injury codes for assault, sexual assault, neglect, abandonment, and maltreatment [4]. These are grouped together and reported as “interpersonal violence” [1,2].

Interpersonal violence includes acts of violence and intimidation that occur between family members, between intimate partners, or between individuals, whether or not they are known to one another, and where the violence is not specifically intended to further the aims of any group or cause [5].

A complex interaction of physiological, psychological, and environmental factors provokes violent behavior. From a public health approach, an ecological model is commonly used to determine risk factors for interpersonal violence [6]. The model considers factors at the individual, interpersonal, community, and societal levels: At the individual level, gender and age are key risk factors. One’s age and gender are associated with a greater or lesser propensity for involvement in interpersonal violence. For example, vulnerability to interpersonal violence changes over the life cycle; youth have the highest propensity to perpetrate or be victimized by interpersonal violence, but the elderly are also vulnerable to interpersonal violence. Young men are most vulnerable as both perpetrators and victims of interpersonal violence [7]. Alcohol or substance use is another major risk factor that influences interpersonal violence at an individual level [1]. The interpersonal level focuses on an individual’s relationship with peers, intimate partners, and family members. The community level examines and seeks to identify settings/locations with a high prevalence of interpersonal violence and the characteristics of these locations that increase the risk of interpersonal violence. Lastly, the societal level looks at the cultural norms, gender, and economic inequality that give rise to interpersonal violence in society [1,7].

About 4400 people die every day because of intentional acts of self-directed, interpersonal, or collective violence. Many thousands more are injured or suffer other non-fatal health consequences because of being either victim or witness to acts of violence. Additionally, tens of thousands of lives are destroyed, families are shattered, and huge costs are incurred in treating victims, supporting families, repairing infrastructure, prosecuting perpetrators, or as a result of lost productivity and investment [1]. In the 53 countries of the WHO European Region, violence kills about 160,000 people each year, and of these, around 31,000 die from interpersonal violence [8].

Deaths are just the tip of the iceberg, and for every death there are numerous admissions to hospital and emergency departments. The clinical pyramid for all types of violence has not been properly estimated, but interpersonal violence is thought to result in at least one million injuries severe enough to warrant medical attention, resulting in high costs, and competing for already over-stretched resources [1].

Injuries and violence are significant causes of death and disease in all countries. They are not evenly distributed across or within countries—some people are more vulnerable than others depending on the conditions in which they are born, grow, work, live, and age. For instance, in general, being young, male, and of low socioeconomic status all increase the risk of injury and of being a victim or perpetrator of serious physical violence [9].

Still, to understand the population burden of exposure to violence and the sub-populations who are at elevated risk, it is important to know the prevalence and socio-demographic factors associated with exposure to interpersonal violence among both men and women [10,11,12].

Most research on the prevalence of interpersonal violence has focused on violence against women and men’s perpetration, in part because of the greater burden of certain forms of violence for women and the greater adverse effects of violence on women’s mental and physical health [13,14,15].

But it is also important to know the impact of other types of violence with long-term consequences for individuals and society, such as child or elder maltreatment. These types of violence often occur in the home and remain hidden because they are rarely reported to the authorities, but victims can access healthcare systems to receive help.

Global policies state the urgent need to address domestic violence and abuse. This problem demands a complex inter-sectoral approach underpinned by a strong universal health system capacity to identify and tailor responses to the circumstances of affected families. The World Health Organization has identified the crucial role of an effective health system in reducing the extensive damage from violence, especially for children [15,16,17,18].

General practice, antenatal clinics, and community child health and emergency departments are key places for interventions against violence, as health practitioners are the major professional group to whom patients want to disclose [19]. Only a minority of women, men and/or children exposed to violence and abuse are recognized in healthcare settings [18]. However, we know that patients want to be asked directly about violence by supportive practitioners, typically making multiple visits before disclosure [19]. Unfortunately, when the patients do disclose, there is evidence that health professionals often lack the essential skills and experience to respond appropriately [17]. Much less is known about the health practitioners’ capacity to identify and respond to children exposed to violence or to men who experience or use violence in their intimate relationship [20,21].

Therefore, healthcare professionals in emergency departments are crucial to detect violence and provide information on the nature of an injury, how it was sustained, and when and where the incident occurred. They are the first step in a healthcare system to obtain more knowledge about both the prevalence and health consequences of interpersonal violence, and as a consequence should have adequate training to suspect violence [22].

As part of the adequate training, it is important to carry out surveys/registries that can provide detailed information about the victim or perpetrator when visiting emergency departments, as well as their background, attitudes, behaviors, and possible previous involvement in violence. Such sources can also help uncover violence that is not reported to the police or other agencies, and can provide valuable information to prevent violence [23].

Our hospital has, since 2009, opened an official registry to report violence or abuse when healthcare professionals suspect them. We aim to share the information collected in the registry throughout these years to expand the available knowledge and implement preventive strategies. Our aim in this article is to assess healthcare professionals’ recognition of types of interpersonal violence among patients admitted to the hospital, including both children and adults, men and women, and factors associated with exposure to violence.

## 2. Materials and Methods

Since 2009 an internal registry of interpersonal violence is conducted in “Hospital Clinico Universitario San Carlos” (third level hospital), located in Madrid.

As a part of routine practice in all hospital departments, an approved questionnaire focused on abuse/violence is administered to admitted patients with suspected interpersonal violence; the questionnaire was approved by the health authorities in the hospital in 2009. The source of information for this study was the data extracted from the questionnaire since 2009 to 2022.

All admitted patients with a suspicion of violence were included; there were no exclusion criteria regarding age or gender.

The questionnaire included the following variables: age, gender, age, country of birth, race, date of abuse/violence, date of medical attention, violence background, reason for consultation, attending specialist, risk factors, vulnerability of the victim, disability, cognitive impairment, current pregnancy, psychiatric diseases, physical or psychiatric dependence, relationship with the perpetrator, coexistence with perpetrator, type of violence (physical violence, sexual violence, economic violence, negligence, chemical submission, mutilation, harassment, other, or combinations of violence), previous assaults, companion, current diagnosis, date of the injury report, chain of custody for obtaining biological samples, previous contact with authorities, assessment by a forensic doctor, social services intervention, healthcare personnel who carried out the registration, attending healthcare professional, referring professional, judicial protection measures, protective measures in the hospital.

The descriptive analyses were assessed. The numerical variable was given a mean ± standard deviation, and the categorical variable was given a frequency. Student’s *t*-test and the Mann–Whitney test were used to compare the means (of quantitative variables) and the Chi-square test of proportions (of categorical variables). A *p*-value of 0.05 was considered statistically significant. Analyses were performed with statistical software XLSTAT version 2023.2.1413 Lumivero (2024), XLSTAT statistical and data analysis solution, New York, NY, USA. https://www.xlstat.com/es; Accessed 15 December 2024.

## 3. Results

The questionnaire was completed by a total of 1284 patients suspected of suffering interpersonal violence. Most of the suspicions (1070) occurred in the emergency department (83.4%), 107 (8.4%) during hospitalization, and 105 (8.2%) in external consultations.

From 2009 to 2022, a total of 1,600,000 patients were admitted to the emergency department, and interpersonal violence was suspected in 0.06%; in outpatient clinics (12,320,000 patients) the percentage of suspicions was 0.0008%, while among the hospitalized patients (420,000 patients) the percentage was 0.02%.

Of the total patients, 80.8% were women and 19.2% were men. The mean age was 33.19 years old (range 0–99; SD 19.83). Subjects were stratified into groups: 13.9% < 18 years old; 75.9% in the group 18–64 and 10.2% in the group ≥ 65 years old.

The distribution between each age group is shown in Table 1. Most of the patients are in group 16–30 years old: 45.2%, followed by the group 31–45 with 26.3%.

Regarding the place of birth, 62.2% (786) were Spanish, followed by patients from South America 19.7% (249), and patients from other European countries (6.2%). The remaining % includes people from other countries such as Africa (3.7%), Central America (3.5%), Antilles (2.3%), Asia (1.3%), North America (1%), and Oceania (0.2%).

**Figure 2:**
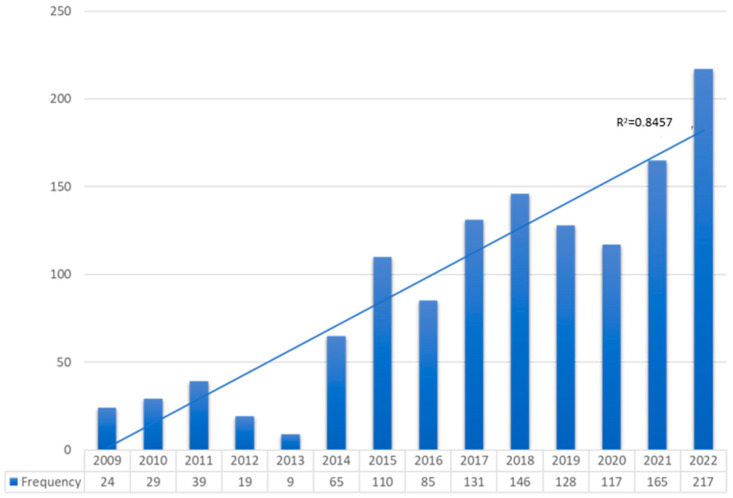
Distribution between years.

A growing number of patients suffering from violence was reported every year since 2009, showing a linear correlation (R^2^ = 0.8457); 16.9% (217) were reported in 2022, which clearly contrasts with the number in 2009, 24 patients (1.9%). The distribution between the years is shown in Figure 2.

When analyzing violence risk factors such as gender, we observed that most of the patients were women (80.8%), and 5.6% (58) of them were pregnant; 18.9% (243) of patients were below 18 or over 80 years old; 10.2% had a disability or were physical dependent; and 11.3% had cognitive impairment, depression or other psychiatric diseases.

In terms of the patients’ relationship with the aggressor, 568 patients (44.2%) did not have any relationship with the perpetrator, while 55.8% of patients did—in 25.2% of cases they were couples, 14.7% (189) received maltreatment by other family members, in 8.2% the perpetrators were acquaintances or friends with the victim, 6.5% of the victims received violence from their ex-partners, and 1.2% from their caregivers.

Surprisingly, 29.4% of patients lived with their aggressor, 3.8% lived and were accompanied to the consultation by their aggressor, and 66% of patients did not maintain any contact with the perpetrator.

Physicians were the health professionals who most frequently completed the registry at 47.6% (607 registries), followed by social workers at 36.6%, psychologists at 12.7% of cases, and nurses at 3.1%. In 65.5% of cases, an injury report was issued.

A description of the types of violence is specified in Table 2. A total of 44.5% of victims suffered physical violence, 22.4% (288 patients) suffered from sexual violence (in 12.8% of cases from an unknown person and in 9.7% from a known person), 13.3% suffered from psychological violence, and 12.5% from economic violence. Negligence was observed in 67 patients (5.2%). A total of 517 patients (40.3%) suffered from chemical submission and 29 (2.3%) from mutilation.

A combination of multiple different types of violence was observed in 36.3% (466) of patients, of whom 382 (29.8%) suffered two types of violence. The most frequent type of combination was sexual violence with chemical submission (107 patients).

## 4. Discussion

The study employs an approved questionnaire within our hospital aimed at detecting victims of interpersonal violence. Healthcare professionals complete it when they suspect abuse, regardless of whether it is verifiable. Unlike other studies, our questionnaire encompasses a wide range of patients collected over 13 years (2009–2022).

Comparing our results with those from other studies in the literature is challenging, since some studies that collect data from violence registries are focused only on emergency departments [6,24,25,26,27], collect data on both the victims and the perpetrators [6,27,28,29], or gather data through surveys administered over short periods of time [26], focusing on specific population groups, such as young people [25]; consequently, the results may vary considerably.

Moreover, the studies conducted in countries differing significantly from ours make extrapolating results challenging due to social, cultural, gender, and economic differences, that contribute to interpersonal violence in society [7].

The percentage of suspected victims of interpersonal violence across the entire hospital was 0.008%, although when extrapolated to the population treated in the emergency room it rose to 0.06%. We have not found any data on the percentage of suspected interpersonal violence in other hospitals, since there are no similar questionnaires in other similar centers in our setting.

The higher percentage of suspicion in the emergency department suggests that professionals there are better trained to identify violence. This aligns with the studies indicating that the health professionals’ training increases suspicion levels [28]. However, while the literature reviews suggest that enhanced training may improve the professionals’ knowledge and attitudes, evidence regarding its impact on recognizing cases is inconclusive. Further randomized and blind studies are needed to ascertain the training’s effectiveness in detecting interpersonal violence [22].

Our results indicate that over 80% of victims were women, consistent with other studies and official agency reports [15,26]. However, this differs from studies in low and middle-income countries, where men are more frequently victims of aggression [6,24,27].

The mean age of the victims was 33.19 years, with the majority falling in the 16–30 age group, consistent with other studies [27]. A systematic review estimates that the risk of violence is also high between ages 31 and 40 [30], corroborating the data on violence resulting in death in our country, which places the age between 26 and 45 years [29]. However, these data pertain solely to female victims of domestic violence.

Most of the victims were Spanish, followed by patients coming from South America; this is consistent with the series of data on deaths caused by domestic violence provided annually by the Council of the Judiciary in Spain [29]. The socioeconomic status of the victims in relation to their nationality remains unknown, potentially influencing interpersonal violence risk. At the individual level, empirical evidence suggests a link between poverty and higher levels of interpersonal violence [31,32,33,34,35,36], particularly concerning child abuse [1]. However, this relationship is complex and often compounded by economic inequality and economic hardship in areas characterized by significant poverty where wealth serves as a stressor, which, we suspect, contributes to the occurrence of violence.

Policies promoting social awareness to change the norms condoning violence, particularly against women, may explain the decrease in violence numbers over time [32,37]. However, our data reveals a linear correlation in the number of victims of violence over the years, suggesting that prevention efforts are not yielding the expected results. The explanation for this increase could be related to the greater awareness among healthcare professionals [38].

In the sample, most of the aggressions came from people close to the victim, their partner or ex-partner was the aggressor in more than 30% of cases, and in more than 30% the aggressors were relatives, friends, acquaintances, or caregivers. Surprisingly, almost 30% of victims lived with the aggressor, and in a small number of cases the perpetrator even accompanied them to the medical consultation. This fact can be interpretated in several ways. The victims of interpersonal violence may remain in abusive relationships due to their desire to keep their family together for fear of losing their children [39,40], as well as due to insecurity and a lack of family and social support [39,41,42], and due to the fear of being killed [43,44]. In addition to these motives is the constant intimidation and belittlement that the partners carry out, by diminishing the victim as a person, making them feel inferior [45].

In our study, most of the questionnaires were completed by physicians, followed by social workers and psychologists, and to a lesser extent by nurses. This is in contrast to other studies, where the responsibility for the screening was relegated to the triage nurse [26]. A qualitative meta-analysis analyzed the physicians’ barriers when recognizing violence. The most frequent reasons were limited time with patients, lack of privacy, lack of management support, a health system that fails to provide adequate training, policies, response protocols, and resources, and the normalization of violence, as well as the belief that it only presents in certain types of women, or that women will lie or are not reliable [46].

The data presented in Table 2 provides a comprehensive overview of the types of violence experienced by victims. Physical violence emerges as the most prevalent form, affecting 44.5% of victims, which correlates with the data from other countries [47,48,49]. Sexual violence was reported by 22.4% of victims, although less frequently than physical assaults, which remains a critical concern. This aligns with our results, where sexual assault by bodily force was reported, with women being more frequently victimized than men [47]. For instance, one study highlighted that one in three women experience sexual assault or violence caused by their intimate partner in their lifetime, and many of these cases are seen in the emergency departments [50]. Emergency rooms face numerous challenges when managing sexual violence cases, including the need for immediate medical and psychological support, forensic evidence collection, and ensuring patient confidentiality [51]. Psychological and economic violence were also notable in our study; we did not find data from other studies to compare the results, but it is clear that the long-term effects of psychological and economic violence on the survivors are profound, impacting both mental and physical health. Psychological violence, often manifesting as emotional abuse, can lead to chronic mental health issues. Negligence was observed in 5.2% of cases. This form of violence, often overlooked, can be particularly damaging, especially among vulnerable populations such as children and the elderly. Addressing negligence requires an increased awareness and stronger policies to ensure adequate care and protection for those at risk. A particularly alarming finding is that 40.3% of victims suffered from chemical submission, indicating a widespread issue of drug-facilitated violence. The high prevalence suggests an urgent need for stricter regulations on substances commonly used in such crimes, as well as improved forensic capabilities to detect and prosecute perpetrators.

There is a significant issue with the underreporting of violence in the healthcare system, which hinders effective management and prevention strategies [52]. Additionally, the absence of validated risk assessment tools complicates the early identification of potential violence [53].

While the above points highlight the common types of interpersonal violence reported in our hospital, it is essential to consider the broader context of these incidents. Factors such as socioeconomic status, cultural norms, and regional policies can influence the prevalence and reporting of violence. Additionally, the underreporting and misclassification of certain types of violence, such as intimate partner violence, suggest that the actual prevalence may be higher than reported. Addressing these issues requires comprehensive public health strategies and improved data collection methods to better understand and mitigate the impact of interpersonal violence globally.

Limitations of this study are the nature of the data, which could be influenced by the training received by professionals over time. In addition, the questionnaire has not been modified over the years, so the evolution in the recognition of interpersonal violence has not been recorded.

## 5. Conclusions

Routine screenings of patients suspected of being the victims of interpersonal violence in a hospital could increase sensitivity to the needs of those who have experienced abuse.

A routine questionnaire could increase the healthcare professionals’ awareness. The level of suspicion of interpersonal violence must be increased in all hospital departments, in addition to the emergency rooms.

Well-trained professionals are crucial to prevent violence and provide safety for the victims of abuse.

Detecting interpersonal violence in a healthcare setting is a critical task that requires a multifaceted approach, integrating advanced technologies, structured screening protocols, and comprehensive data analyses.

The complexity of hospital environments, combined with the often subtle presentation of violence-related injuries, necessitates innovative strategies to effectively identify and address these cases.

Injury prevention programs can then be instituted in the community with the collaborative efforts of healthcare providers.

## Figures and Tables

**Table 1:** Age group distribution.

Age	Frequency	Percent	Valid percent	Cumulative Percent
<1 year	14	1.1	1.1	1.1
1–15	122	9.5	9.5	10.6
16–30	578	45.0	45.2	55.8
31–45	337	26.2	26.3	82.1
46–50	49	3.8	3.8	85.9
51–65	52	4.0	4.1	90.0
66–80	66	5.1	5.2	95.2
>80	62	4.8	4.8	100
Total	1280	99.7	100	
Missing values	4	0.3		
Total	1284	100.0		

**Table 2:** Type of violence distribution.

Type of Violence		Frequency	Percent
Physical Violence		572	44.5
Sexual Violence	Known aggressor	124	9.7
	Unknown aggressor	164	12.8
Psychological Violence		171	13.3
Economic Violence		160	12.5
Negligence		67	5.2
Chemical submission		517	40.3
Mutilation		29	2.3
Others		37	2.9

## Data Availability

The original contributions presented in this study are included in the article. Further inquiries can be directed to the corresponding author.
